# Impacts of Antimalarial Drugs on *Plasmodium falciparum* Drug Resistance Markers, Western Kenya, 2003–2015

**DOI:** 10.4269/ajtmh.17-0763

**Published:** 2018-01-22

**Authors:** Elizabeth Hemming-Schroeder, Emuejevuoke Umukoro, Eugenia Lo, Becky Fung, Pedro Tomás-Domingo, Guofa Zhou, Daibin Zhong, Amruta Dixit, Harrysone Atieli, Andrew Githeko, Anne Vardo-Zalik, Guiyun Yan

**Affiliations:** 1 Program in Public Health, University of California, Irvine, California;; 2 Department of Biological Sciences, University of North Carolina, Charlotte, North Carolina;; 3 Centre for Vector Biology and Control Research, Kenya Medical Research Institute, Kisumu, Kenya;; 4 Department of Biology, Penn State York, York, Pennsylvania

## Abstract

Antimalarial drug resistance has threatened global malaria control since chloroquine (CQ)-resistant *Plasmodium falciparum* emerged in Asia in the 1950s. Understanding the impacts of changing antimalarial drug policy on resistance is critical for resistance management. *Plasmodium falciparum* isolates were collected from 2003 to 2015 in western Kenya and analyzed for genetic markers associated with resistance to CQ (*Pfcrt*), sulfadoxine–pyrimethamine (SP) (*Pfdhfr*/*Pfdhps*), and artemether–lumefantrine (AL) (*PfKelch13*/*Pfmdr1*) antimalarials. In addition, household antimalarial drug use surveys were administered. *Pfcrt* 76T prevalence decreased from 76% to 6% from 2003 to 2015. *Pfdhfr*/*Pfdhps* quintuple mutants decreased from 70% in 2003 to 14% in 2008, but increased to near fixation by 2015. SP “super resistant” alleles *Pfdhps* 581G and 613S/T were not detected in the 2015 samples that were assessed. The *Pfmdr1* N86-184F-D1246 haplotype associated with decreased lumefantrine susceptibility increased significantly from 4% in 2005 to 51% in 2015. No *PfKelch13* mutations that have been previously associated with artemisinin resistance were detected in the study populations. The increase in *Pfdhfr*/*Pfdhps* quintuple mutants that associates with SP resistance may have resulted from the increased usage of SP for intermittent preventative therapy in pregnancy (IPTp) and for malaria treatment in the community. Prevalent *Pfdhfr*/*Pfdhps* mutations call for careful monitoring of SP resistance and effectiveness of the current IPTp program in Kenya. In addition, the commonly occurring *Pfmdr1* N86-184F-D1246 haplotype associated with increased lumefantrine tolerance calls for surveillance of AL efficacy in Kenya, as well as consideration for a rotating artemisinin-combination therapy regimen.

## INTRODUCTION

Antimalarial drug resistance has significantly hindered malaria control efforts and played a key role in shaping global drug policies since the first reports of chloroquine (CQ) resistance arose from Southeast Asia in 1957.^1^ Since then, because of widespread drug resistance, global recommendations for the first-line treatment of malaria have changed from CQ to sulfadoxine–pyrimethamine (SP), and again, most recently, from SP to artemisinin-combination therapy (ACT).^1^ As both CQ and SP drug resistance arose in Southeast Asia before spreading to Africa,^2^ the emergence of ACT resistance in several Southeast Asian countries and recent report on the emergence of indigenous artemisinin-resistant *Plasmodium falciparum* in Africa^3^ triggers major concern on the efficacy of malaria control programs in Africa where most of the global malaria burden falls.^4^

Although delayed clearance of the parasite following artemisinin treatment has been reported in African countries, such as Kenya,^5^ Nigeria,^6^ and Angola,^7^ the association of African *PfKelch13* mutations with clinical resistance is not clear and mutations associated with artemisinin resistance in Southeast Asia have yet to be commonly observed in Africa.^8–12^ Close monitoring and resistance validation of *PfKelch13* mutations, as well monitoring for mutations associated with ACT partner drug resistance in East Africa will be critical to detecting the spread of ACT resistance from Southeast Asia to Africa or indigenous emergence. In addition, although ACTs have been implemented as first-line treatment of malaria in Kenya since 2006, other antimalarial drugs, including SP and CQ, continue to be used for treating malaria,^13^ further complicating malaria treatment in Kenya. Moreover, intermittent preventative treatment of malaria in pregnancy (IPTp) with SP as prophylaxis for malaria in pregnancy was adopted as the Kenyan national policy in 1998,^14^ which may lead to continued selection pressure for mutations associated with SP resistance.

Here, we investigated the dynamics of antimalarial drug resistance markers in response to changing antimalarial drug policy in western Kenya. *Plasmodium falciparum* samples across the years 2003, 2005, 2008, and 2015 were examined, before and after the first mass distribution of artemether–lumefantrine (AL) in Kenya in 2006. Frequencies of amino acid polymorphisms in genes including *Pfcrt* for CQ resistance,^2^ *Pfdhfr* and *Pfdhps* for SP resistance,^15^ *Pfmdr1* for lumefantrine tolerance,^16^ and *PfKelch13* for artemisinin resistance^17^ were assessed. We examined whether the observed amino acid changes have been undergoing selection through a longitudinal comparison of mutation frequencies in these drug resistance genes. Understanding the impacts of the antimalarial drug policy on molecular markers of drug resistance and monitoring for artemisinin resistance are critical to informing antimalarial drug policy in Kenya.

## METHODS

### Study design and participants.

This study was conducted in two sites in western Kenya: Kakamega (0.282° N, 34.752° E), a low malaria-transmission site, and Kombewa (0.105° S, 34.520° E), a high malaria-transmission site. The differences in malaria transmission intensities is partly attributed to the differences in altitude between sites, where Kakamega is in the highlands (1,430–1,580-m elevation) and Kombewa is a lowland site (1,170–1,300 m). Blood samples were collected from asymptomatic school children between the ages of 6–15 years in 2003, 2005, 2008, and 2015. Sampling methods were consistent across the years studied. School-aged children were studied because they are among the age groups with the highest risk of malaria infection. A total of 705 *P. falciparum* isolates were collected between 2003 and 2015 at the two study sites, ranging from 29 to 194 isolates per site per year (Supplemental Table 1). Samples with more than one mixed (mutation/wildtype) mutation site were discarded from haplotype analyses, but were included in individual single nucleotide polymorphism (SNP) analyses. Blood dots were made on a filter paper for genotyping and stored at −20°C until use.

Scientific and ethical clearance was given by the institutional scientific and ethical review boards of the Kenya Medical Research Institute, Kenya, and the University of California, Irvine, CA. Written informed consent/assent for study participation was obtained from all consenting heads of households and each individual who was willing to participate in the study.

### Procedures.

The Saponin/Chelex method was used to extract parasite DNA from dried blood samples.^18^ Quantitative polymerase chain reaction (PCR) of *P. falciparum*-specific 18S rRNA was used to detect *P. falciparum* infections.^19^ *Plasmodium falciparum* isolates were genotyped at *Pfcrt* for CQ resistance; *Pfdhfr* and *Pfdhps* for SP resistance; *Pfmdr1* for lumefantrine tolerance; and *PfKelch13* for artemisinin resistance (see Supplemental Table 2 for codon positions). For genes *Pfcrt*, *Pfmdr1*, *Pfdhfr*, and *Pfdhps*, a restriction enzyme digestion protocol was used to detect specific mutations among samples collected in 2003, 2005, and 2008,^20^ and a subset of mutations were confirmed by direct sequencing; for samples in 2015, the mutations of these target genes were assessed by PCR and sequencing (Supplemental Table 2).^21,22^ For *PfKelch13*, samples were amplified and sequenced using the published protocol^17^ with modifications (Supplemental Table 2). We used the *Pfcrt* 76T mutation as a proxy for CQ resistance and *Pfdhfr*51I-59R-108N/*Pfdhps*437G-540E quintuple mutant for SP resistance because of their strong associations with antimalarial resistance.^2,15^ In addition, SP “super resistant” alleles *Pfdhps* 581G, *Pfdhps* 613S/T, and *Pfdhfr* 164L were examined in 2015 isolates.^15^ The *Pfmdr1* N86-184F-D1246 haplotype was used as a proxy for reduced AL susceptibility because of evidence that lumefantrine selects for this haplotype.^23–25^ Nonsynonymous mutations in the *PfKelch13* propeller region were assessed for artemisinin resistance, given that single amino acid changes in this region have been associated with in vivo and ex vivo resistance.^8,17,26,27^ Amplified PCR fragments were purified and sequenced from both ends by Sanger sequencing (GENEWIZ, Inc., South Plainfield, NJ). All sequences were blasted against NCBI GeneBank database for verification. Sequences were visualized using Chromas v2.5.0, aligned with ClustalX v2.1, and manually edited in Bioedit v7.2.5. Sequences were deposited to Genbank (accession numbers MF344967–MF345825).

### Household antimalarial usage surveys.

A cross-sectional survey was conducted for a total of 10,519 randomly selected households in western Kenya in the years 2003, 2007, 2011, and 2016 to assess antimalarial drug usage. For the years 2003 and 2007, surveys were conducted in Kakamega and Kisii counties, and in 2010 and 2016, surveys were conducted in Kakamega and Vihiga counties. No significant differences between sites within years were observed, and so results from multiple sites were pooled for visualization and analysis. Questionnaires were administered to an adult member of each surveyed household. Specifically, in the questionnaires, household heads were asked to name which medicine was used for the family member who had the most recent malaria episode.

### Data analysis.

Two-tailed χ^2^ tests and Fisher’s exact tests were conducted to make pairwise comparisons for mutation frequencies between sites and years for all haplotypes and individual polymorphisms assessed. A Bonferonni correction for 28 tests was applied, placing significance at 0.0018. The 95% confidence intervals were computed using the binomial distribution. Linkage disequilibrium was tested for all samples with complete genotypes after omitting samples from mixed infections (*N* = 168). Linkage disequilibrium estimates were calculated in Genepop 4.2 for all possible pairs of loci.^28^ *P* values were calculated using Fisher’s tests with a Bonferonni correction for 36 tests across nine loci, placing significance at 0.0014.

## RESULTS

**Figure 1.**
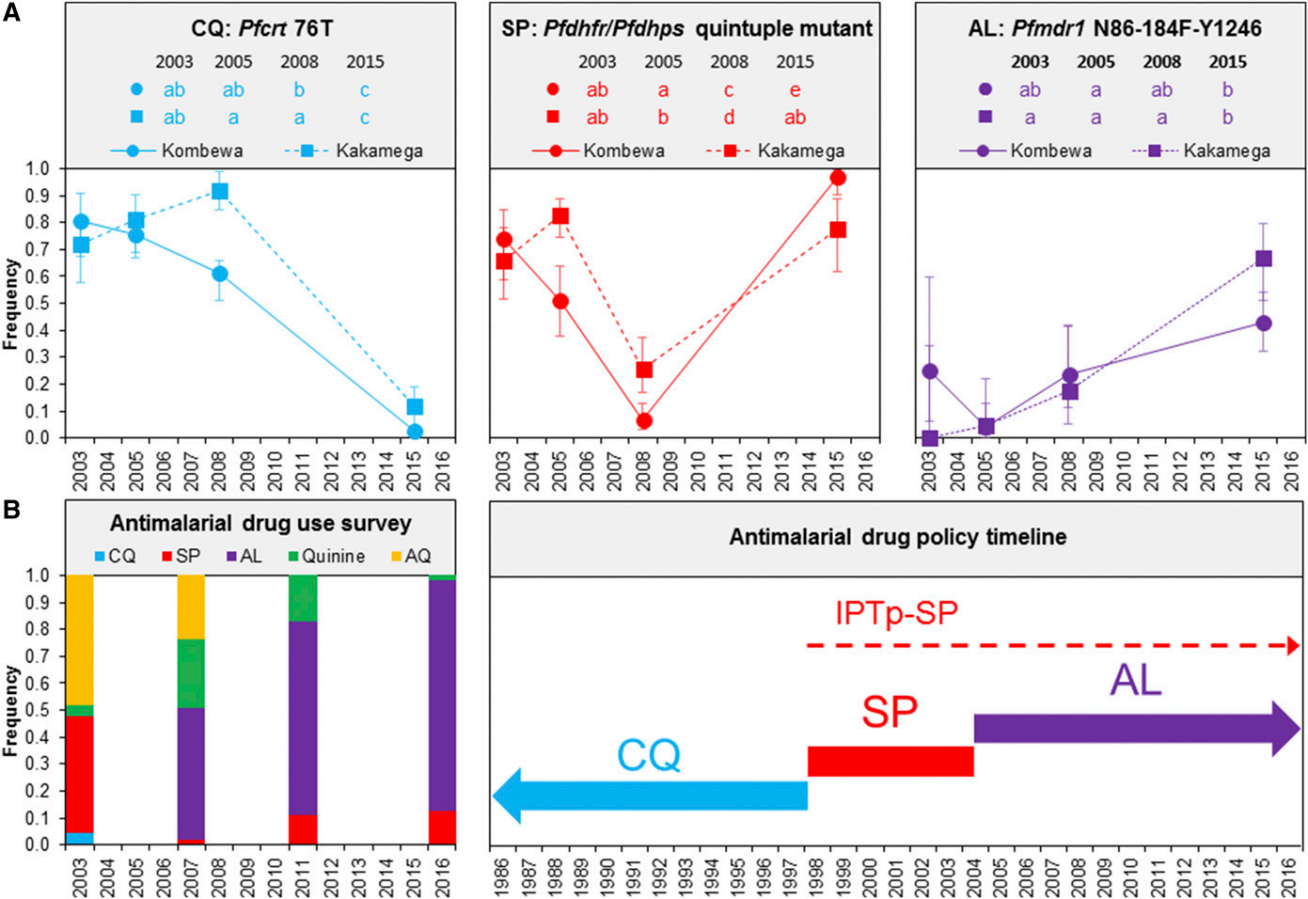
A comparison of (**A**) *Plasmodium falciparum* drug resistance molecular markers to (**B**) reported antimalarial drug usage for treatment and antimalarial drug policy timeline in western Kenya. AL = artemether–lumefantrine; AQ = amodiaquine; CQ = chloroquine; SP = sulfadoxine–pyrimethamine. *Pfhfr*/*Pfdhps* quintuple mutant is *Pfdhfr*51I-59R-108N/*Pfdhps*437G-540E. Error bars represent 95% confidence intervals. Shared lowercase letters between study sites/years indicate that they are not significantly different from each other. Differing lowercase letters indicate statistically significant differences between study sites/collection years. Statistical significance was determined from the results of Fisher’s exact tests with a Bonferroni correction for 28 tests between study sites and years (*P* < 0.0018). The timelines for first-line antimalarial drugs are indicated by bold arrows, whereas the timeline for intermittent preventative therapy in pregnancy (IPTp) is indicated by the dashed arrow. This figure appears in color at www.ajtmh.org.

Significant changes in frequencies of drug resistance molecular markers were observed with changes in antimalarial drug policy and reported use over the 13-year study period in western Kenya. A decreasing trend in *Pfcrt* 76T mutation, associated with CQ resistance,^2^ was observed from 2003 to 2015 at both study sites (Figure 1A). However, differences were observed between the sites in 2008 when the *Pfcrt* 76T mutation was observed at a significantly higher frequency in Kakamega at 91.9% than in Kombewa at 61.0%. By 2015, *Pfcrt* 76T mutation frequencies declined to 2.7% and 11.8% in Kombewa and Kakamega, respectively.

**Figure 2.**
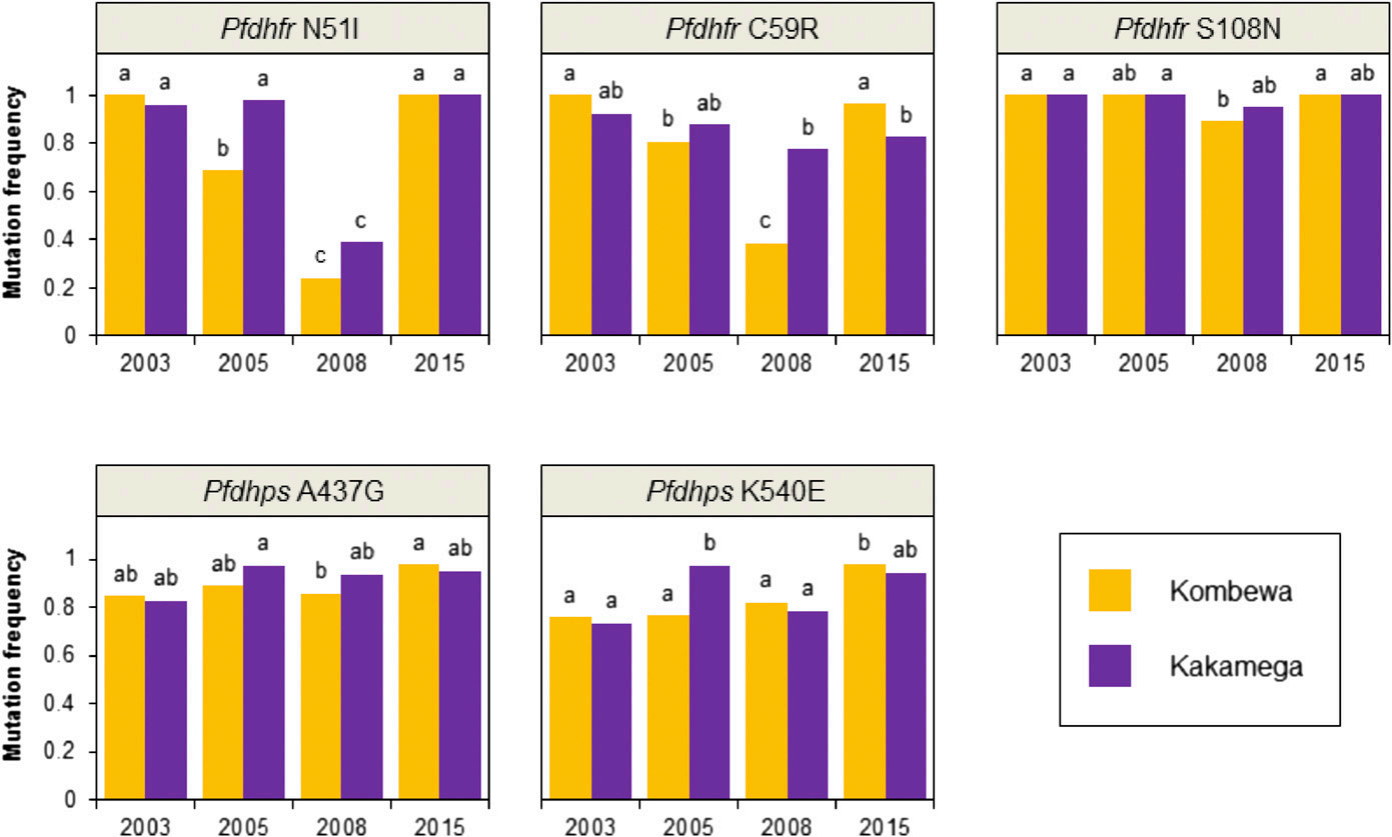
Mutation frequencies for genetic markers associated with sulfadoxine–pyrimethamine resistance in *Plasmodium falciparum* samples collected in 2003–2015 at Kombewa and Kakamega. Differing lowercase letters indicate statistically significant differences between study sites/collection years. Statistical significance was determined from the results of Fisher’s exact tests with a Bonferroni correction for 28 tests (*P* < 0.0018). This figure appears in color at www.ajtmh.org.

Individual mutations important for SP resistance^15^ revealed varying trends over the years at both study sites (Figure 2). For instance, the frequency of *Pfdhfr* N51I mutation decreased significantly between 2003 and 2008, but rebounded and increased significantly in 2015 (Figure 2). Likewise, a significant decrease in *Pfdhfr* C59R mutation was detected at Kombewa from 2003 to 2008, but the mutation frequency bounced back in 2015 to a high level as seen in 2003 (Figure 2). Whereas, for *Pfdhfr* S108N, the most important mutation for in vitro pyrimethamine resistance,^2^ there was relatively little change in mutation frequencies across the years at both study sites (Figure 2). *Pfdhps* A437G and *Pfdhps* K540E showed a similar trend of little change across the years (Figure 2). The *Pfdhfr* triple mutant and *Pfdhps* double mutant were also found to be most prevalent haplotypes among the 2015 samples (Supplemental Table 3). The “super resistant” alleles *Pfdhps* 581G and *Pfdhps* 613S/T were not detected in 2015 samples, which was also the result of a 2005 study in western Kenya.^29^ The “super resistant” allele *Pfdhfr* 164L was detected in one 2015 isolate (Supplemental Table 3).

For *Pfdhfr*/*Pfdhps* haplotype analysis, a total of 300 samples were excluded for having either multiple mixed mutation sites or incomplete haplotypes (see Supplemental Table 4 for frequencies of mixed polymorphisms). Although the quintuple *Pfdhfr*/*Pfdhps* mutant, considered to be fully resistant to SP,^15^ decreased between 2005 and 2008 following the policy change to AL in 2004 (although AL was not distributed until 2006) (Figure 1B), both study sites experienced a significant increase in *Pfdhfr*/*Pfdhps* quintuple mutants between 2008 and 2015, exceeding the frequencies seen in 2003 (Figure 1A). For instance, at Kombewa, *Pfdhfr*/*Pfdhps* quintuple mutant frequencies increased by 15-fold from 2008 to 2015; a 3-fold increase was also found in Kakamega within the same time period. By 2015, *Pfdhfr*/*Pfdhps* quintuple mutant frequencies were 96.7% at Kombewa and 77.8% at Kakamega.

**Figure 3.**
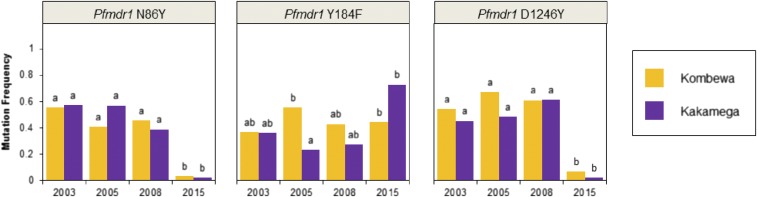
Mutation frequencies for genetic markers associated with lumefantrine resistance in *Plasmodium falciparum* samples collected in 2003–2015 at Kombewa and Kakamega. Differing lowercase letters indicate statistically significant differences between study sites/collection years. Statistical significance was determined from the results of Fisher’s exact tests with a Bonferroni correction for 28 tests (*P* < 0.0018). This figure appears in color at www.ajtmh.org.

*Pfmdr1* N86, 184F, and D1246 polymorphisms are associated with decreased lumefantrine susceptibility,^16,23–25^ although N86 may be the most important polymorphism for increased lumefantrine tolerance.^16,24^ A significant decrease in mutation prevalence was observed from 2008 to 2015 for *Pfmdr1* N86Y and *Pfmdr1* D1246Y at both study sites. Whereas, for *Pfmdr1* Y184F, at Kakamega, an increase in mutation frequencies was observed from 2005 to 2015. At Kombewa, there was no significant change in *Pfmdr1* Y184F mutation frequencies across collection years (Figure 3).

For *Pfmdr1* haplotype analysis, a total of 154 samples were excluded for having either multiple mixed mutation sites or incomplete haplotypes (see Supplemental Table 4 for frequencies of mixed polymorphisms). A significant increase in the *Pfmdr1* N86-184F-D1246 haplotype frequency, associated with lumefantrine tolerance,^23–25^ was observed at Kombewa and Kakamega between 2005, when frequencies were 4.2% and 4.5%, respectively, and 2015. By 2015, the *Pfmdr1* N86-184F-D1246 frequency at Kombewa was 42.7% and at Kakamega was 66.7%. Estimates of linkage disequilibrium revealed no significant linkage between any of the polymorphisms investigated. Although not statistically significant after applying a Bonferonni correction, locus pairs *Pfdhps* 437/*Pfdhps* 540 and *Pfdhfr* 59/*Pfdphs* 540 were the most closely linked (*P* = 0.006 and *P* = 0.028, respectively).

**Figure 4.**
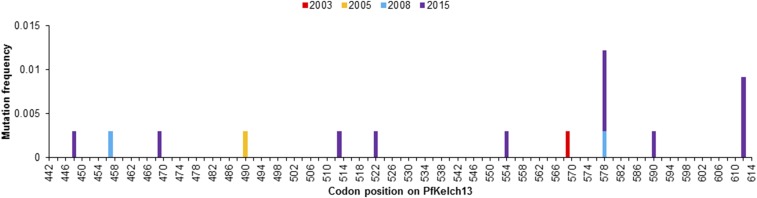
Nonsynonymous *PfKelch13* mutations in western Kenya, 2003–2015. This figure appears in color at www.ajtmh.org.

Eleven unique nonsynonymous mutations were observed in *PfKelch13* among our samples (Figure 4, Supplemental Table 5). The most common mutations were A578S that was found in four isolates and E612D in three isolates (Figure 4). The remaining nine mutations were observed individually in only one isolate: I448M, L457I, C469W, N490S, R513S, S522C, A554S, A569S, and I590F.

According to the self-reported antimalarial drug use for treatment household survey (Figure 1B), SP drug use was less than 2% in 2007, 1 year after the distribution of AL. Nevertheless, the SP drug use increased to 10.8% in 2011 and 12% in 2016, despite the fact that AL was increasingly used as the first-line treatment from 2007 (49.2%) to 2016 (81%; Figure 1B). CQ use was at 4.8% in 2003, but was not reported in any of the following years. Antimalarial drug use surveys also revealed that in 2016, 92% of women who were pregnant in the past 4 months (*N* = 109) took at least one dose of SP as IPTp while pregnant (Table 1). This level of IPTp-SP coverage increased from previous years in western Kenya, when coverage was 16% in 2001^30^ and 51% in 2008.^31^

**Table 1:** Intermittent preventative therapy in pregnancy (IPTp) antimalarial drug usage in western Kenya (*n* [%])

	2001 (*n* = 903)	2008 (*n* = 444)	2016 (*n* = 109)
Took at least one dose of IPTp-SP while pregnant	147 (16%)	227 (51%)	100 (92%)
Communities surveyed	Kisii, Bondo	Kisii, Bondo	Kakamega, Vihiga
Data source	Guyatt et al. (2004)^30^	Gikandi et al. (2008)^31^	This study

SP = sulfadoxine–pyrimethamine. *n* is the total number of pregnant women surveyed.

## DISCUSSION

This study examined the impact of past and present antimalarial drug policy and usage on drug resistance genetic markers of *P. falciparum*, the most common and deadly malarial parasite in sub-Saharan Africa.^4^ We found that mutations associated with resistance have declined for CQ, but have increased for SP following an initial decline. In addition, the prevalence of polymorphisms associated with lumefantrine tolerance has increased since pre-AL distribution levels. No known mutations associated with artemisinin resistance in Asia were detected.

CQ was retracted as first-line antimalarial treatment in 1998 because of increasing and widespread reports of CQ resistance in sub-Saharan Africa.^32^ Its resistance is primarily attributed to the mutation *Pfcrt* K76T.^2^ As expected, we observed a significant decline in *Pfcrt* K76T prevalence to very low levels by 2015. Our findings corroborated the results from the community surveys that indicated very low CQ usage for antimalarial treatment over the past 12 years. Reduced selection pressure might no longer favor CQ resistance mutations, and thus a drastic reduction in mutants was observed from 2003 to 2015. In addition, AL has been demonstrated to select for CQ-susceptible parasites.^33^ Thus, the observed increase in AL coverage could also favor the wildtype *Pfcrt* polymorphism. The decline of *Pfcrt* K76T mutation in Kenya, which was also reported in previous studies,^32,34,35^ as well as nearby countries Tanzania^36^ and Rwanda,^37^ calls for careful study into the possibility for CQ to be reintroduced, such as in a combination therapy or in limited cases. However, the risk of rapid reemergence of CQ resistance should be cautiously evaluated before a potential reintroduction.

SP replaced CQ as the first-line treatment of malaria in Kenya in 1999. However, by 2003, reports showed that SP effectiveness was also faltering.^38^ Quintuple mutants consisting of *Pfdhps* 437/540 and *Pfdhfr* 51/59/108 are considered to be fully resistant to SP.^15^ These quintuple mutants were present in 91.3% of the isolates collected in 2015, which was remarkably higher than that observed in 2008 (13.8%). The striking increase in *Pfdhps*/*Pfdhfr* mutants could be partly explained by the observed increase in SP usage from less than 2% in 2007 to 12% in 2016, even though the frequency of SP use in 2016 was still less than that observed in 2003 (45.2%). Another explanation for the drastic rise in quintuple *Pfdhps*/*Pfdhfr* mutants could be the increased use of SP as IPTp, a guideline put forth by the World Health Organization (WHO).^39^ IPTp with SP as prophylaxis for malaria in pregnancy was adopted as the Kenyan national policy in 1998.^14^ IPTp coverage was low in the first few years, with only 16% of pregnant women in western Kenya reported taking at least one dose of IPTp-SP in 2001.^30^ Since then, the IPTp coverage has been increasing over the years with up to 51% of pregnant women in 2008^31^ and 92% in 2016 (this study) reported taking at least one dose of IPTp-SP. The substantial increase in coverage of IPTp-SP from 2001 to 2016, coupled with the moderate increase in SP usage for malaria treatment from 2007 to 2016 likely impose selection pressure for SP-resistant parasites.

Apart from Kenya, the increasing *Pfdhfr*/*Pfdhps* mutation frequencies have also been reported in several other African countries.^15^ The dominance of fully resistant SP mutants is concerning because this could decrease IPTp-SP effectiveness and exacerbate malaria infections.^40^ Although WHO recommends the continuation of IPTp-SP in malaria-endemic countries across Africa,^39^ it is imperative to monitor its effectiveness given the very high levels of fully resistant mutants observed in this study.^15^ The emergence of “super resistant” alleles, such as *Pfdhfr* 164L, *Pfdhps* 581G, and *Pfdhps* 613S/T sextuple mutant haplotype, may further diminish the effectiveness of IPTp-SP.^15,41,42^ For example, the 581G mutation has been associated with increased parasitemia in pregnant women in Tanzania.^43^ Notably, we did not detect such “super resistant” haplotypes in our study populations despite that they have been previously detected in western Kenya at varying frequencies.^44,45^ Careful monitoring of SP resistance and emergence of “super resistant” alleles is critical.

AL was first distributed in Kenya in 2006, following the policy change from SP to AL for first-line antimalarial treatment. Changes in lumefantrine sensitivity have been associated with polymorphisms in the *Pfmdr1* gene.^16,25^ For example, Tanzanian parasites having the *Pfmdr1* N86-184F-D1246 haplotype were able to withstand lumefantrine blood concentrations 15-fold higher than parasites with the 86Y-Y184-1246Y haplotype.^23^ In addition, in Uganda, AL was demonstrated to select for haplotypes with N86 in combination with 184F, D1246, or both.^24^ Our findings of a significantly increased prevalence of N86-184F-Y1246 haplotypes since before the distribution of AL suggest that this haplotype is being selected for by AL. This finding of a commonly occurring haplotype associated with decreased lumefantrine susceptibility calls for continued surveillance of AL efficacy in Kenya. In addition, as other ACTs such as artesunate–amodiaquine and dihydroartemisinin–piperaquine pose different selective pressures on *Pfmdr1* haplotypes than AL, rotating ACT regimens may be an effective strategy for delaying ACT partner drug resistance in Kenya.^24,46^

No mutations associated with artemisinin resistance in Asia have been observed in our study populations. However, other *PfKelch13* mutations were observed at low frequencies. The nonsynonymous A578S *PfKelch13* mutation observed in this and other studies^8,9,47–49^ was not found to be associated with artemisinin resistance when introduced in the Dd2 line.^8^ The *PfKelch13* E612D mutation observed in the present study has not been examined in regard to its association with AL resistance, but it has been observed in other parts of Africa.^50^ Seven other nonsynonymous mutations were detected in our isolates post-ACT distribution. The fact that none of the Southeast Asian *PfKelch13* mutations were detected in our study populations suggests that there may be a combination of different factors that play a role in artemisinin resistance between the two continents.^47^ For example, artemisinin resistance may require additional mutations at secondary loci,^51^ such as those candidate SNPs identified by Chebon et al.^52^ in Kenyan *P. falciparum* on chromosomes 12 and 14. This notion is underscored by the observance of common delayed clearance of the parasite following ACT treatment in Kenya.^5^ In addition, a 5-year longitudinal study conducted in Uganda found that there was a correlation between the increased usages of ACT in communities with decreased sensitivities of the parasites to the drug.^53^ These results suggest the possibility of an independent emergence of artemisinin resistance in Africa, which is not associated with *PfKelch13* mutations. As a result, closer surveillance of widespread ACT usage and deeper analyses of the parasite genome are needed to identify new or potential markers for artemisinin resistance in Africa.

Our study had certain limitations. The present study was limited to two study sites in western Kenya. It is unclear whether a similar pattern is observed in other parts of Kenya or other countries. We did not examine *Pfmdr1* copy number, which has been shown to be associated with lumefantrine tolerance.^54^ In addition, sample sizes for *PfKelch13* were relatively small especially in 2003 because of limited DNA quantity and quality in some of those earlier samples, which limits our ability to detect rare mutations. Last, Sanger sequencing of PCR products used in the present study has a lower sensitivity in detecting rare mutations in infections with multiple clones compared with deep sequencing methods. However, the overall trends reported here would not be affected by these limitations.

The findings from this study have significant implications for malaria control in Kenya. First, the efficacy of IPTp-SP in Kenya could be diminished by the near fixation of fully resistant SP mutants. Second, approximately 10% of surveyed patients continue to use SP for malaria treatment despite the policy that AL is the recommended first-line drug. Because fully resistant SP mutants are predominant in this region, it is conceivable that these patients experience high malaria treatment failure rates. Third, we found that artemisinin resistance has not yet spread from Southeast Asia to western Kenya as evidenced by the absence of *PfKelch13* mutations in Kenya that are known to be associated with drug resistance in Southeast Asia. However, we detected a nonsynonymous mutation *PfKelch13* E612D in multiple isolates that may be a potential candidate for in vitro validation for artemisinin resistance. Last, we found an increase in *Pfmdr1* haplotypes associated with decreased lumefantrine susceptibility, which calls for continued monitoring of AL effectiveness and potentially implementing multiple first-line ACTs to delay ACT partner drug resistance.

This study sheds light on the long-term dynamics of drug resistance markers in response to antimalarial policy. Our findings suggest that changes in first-line antimalarial treatment and IPTp policies have been followed by dramatic changes in molecular drug resistance markers. In addition, despite policy changes, ineffective drugs continue to be used for extended amounts of time, which may lead to the persistence of drug resistance markers. Understanding the interplay between drug resistance on a molecular level, antimalarial drug usage, and antimalarial drug policy is critical to informing antimalarial drug use policies.

## Supplementary Material

Supplemental Table.
