# Supplementary material for: Impacts of Antimalarial Drugs on Plasmodium falciparum Drug Resistance Markers, Western Kenya, 2003–2015
Source: Am J Trop Med Hyg. 2018 Jan 22;98(3):692–9. doi: 10.4269/ajtmh.17-0763 (PMC5930917; doi:10.4269/ajtmh.17-0763)
Supplement: Supplementary file 1 [file tpmd170763.SD1.pdf]

Supplemental Table 1

Total number of *Plasmodium falciparum* samples collected by gene/site/collection year in western Kenya

	Pfcrt	*Pfmdr1*	*Pfdhfr*	*Pfdhps*	*PfKelch13*
Kombewa					
2003	33	18	33	33	29
2005	61	73	73	73	40
2008	177	165	194	194	35
2015	75	86	90	93	43
Kakamega					
2003	53	28	53	53	32
2005	106	108	109	109	31
2008	62	67	82	82	53
2015	48	41	49	40	66

Supplemental Table 2

Primers used for sequencing of antimalarial drug-resistant markers

Gene	Target codon(s)	Name	Base sequence for primers (3′ to 5′)	References
Pfcrt	76	crt-P1F	GGAGGTTCTTGTCTTGGTAAAT	Zhang et al. (2008)[21]
		crt-P1R	ATATTGGTAGGTGGAATAGATTCT	Zhang et al. (2008)[21]
Pfmdr1	86, 184	pfmdr1a-F	CATTTGTATGTGCTGTATTAT	Designed in this study
		pfmdr1a-R	CCACAAACATAAATTAACGGA	Designed in this study
	1,246	pfmdr1C-F	TGTAAATGAATTTTCAAACCAATC	Designed in this study
		pfmdr1C-R	CCATATGGTCCAACATTTGTAT	Designed in this study
Pfdhfr	51, 59, 108, 164	dhfr-P4F	TGATGGAACAAGTCTGCGACGTT	Zhang et al. (2008)[21]
		dhfr-P4R	CTGGAAAAAATACATCACATTCATATG	Zhang et al. (2008)[21]
Pfdhps	(Primary)	m3717	CCATTCCTCATGTGTATACAA	Wang et al. (1995)[22]
		186	GTTTAATCACATGTTTGCACTTTC	Wang et al. (1995)[22]
	(Nested) 437, 540, 581, 613	pfdhps-F	TTGTTGAACCTAAACGTGCTG	Designed in this study
		pfdhps-R	TCCAATTGTGTGATTTGTCCA	Designed in this study
PfKelch13	(Primary)	K13-1	CGGAGTGACCAAATCTGGGA	Ariey et al. (2014)[17]
		K13-4	GGGAATCTGGTGGTAACAGC	Ariey et al. (2014)[17]
	(Nested) 442–614	K13-2F	TGATCTAGGGGTATTCAAAGGTG	Designed in this study
		K13-2	GCCAAGCTGCCATTCATTTG	Ariey et al. (2014)[17]
		K13-3	GCCTTGTTGAAAGAAGCAGA	Ariey et al. (2014)[17]
		K13-3R	GTGGTGTTACGTCAAATGG	Designed in this study

Supplemental Table 3

Proportion of haplotypes on *Pfdhfr* and *Pfdhps* genes of *Plasmodium falciparum* isolates collected in western Kenya, 2015

Haplotypes	Kombewa	Kakamega
Pfdhfr		
Triple		
51I/59R/108N/164I	0.967	0.827
51I/59C/108N/164L	0.011	0.000
Double		
51I/59C/108N/164I	0.022	0.173
Pfdhps		
Triple		
436P/437G/540E	0.118	0.050
436H/437G/540E	0.086	0.025
Double		
436S/437G/540E	0.774	0.900
436P/437A/540E	0.000	0.025
Wildtype		
436S/437A/540K	0.022	0.000

Mutations are underlined.

Supplemental Table 4

Frequencies of polymorphisms in *Pfcrt*, *Pfmdr1*, *Pfdhfr*, and *Pfdhps* with mixed infections as a third category

			Kombewa				Kakamega			
Gene	Mutation	Polymorphism	2003	2005	2008	2015	2003	2005	2008	2015
Pfcrt	K76T	Mutant	80.6	66.7	50.0	1.8	63.3	76.1	91.2	7.3
		Mixed	6.5	13.9	22.2	1.8	20.0	13.4	2.9	4.9
		Wild-type	12.9	19.4	27.8	96.4	16.7	10.4	5.9	87.8
Pfmdr1	N86Y	Mutant	36.4	21.2	25.3	1.5	46.7	42.0	23.7	0.0
		Mixed	27.3	42.4	39.8	1.5	33.3	31.9	23.7	0.0
		Wild-type	36.4	36.4	34.9	97.0	20.0	26.1	52.6	100
	Y184F	Mutant	38.1	40.0	22.2	33.3	8.0	8.6	28.6	70.6
		Mixed	23.8	30.0	37.0	13.6	56.0	25.9	0.0	0.0
		Wild-type	38.1	30.0	40.7	53.0	36.0	65.5	71.4	29.4
	D1246Y	Mutant	35.5	52.3	37.0	4.6	20.0	31.9	40.0	0.0
		Mixed	32.3	34.1	50.0	4.6	50.0	37.7	53.3	2.7
		Wild-type	32.3	13.6	13.0	90.8	30.0	30.4	6.7	97.3
Pfhdfr	N51I	Mutant	100	59.1	1.0	100	96.7	98.6	21.3	100
		Mixed	0.0	20.5	45.9	0.0	0.0	0.0	29.8	0.0
		Wild-type	0.0	20.5	53.1	0.0	3.3	1.4	48.9	0.0
	C59R	Mutant	100	72.7	22.2	97.1	90.0	85.5	73.9	83.3
		Mixed	0.0	15.9	33.3	0.0	3.3	14.5	4.3	0.0
		Wild-type	0.0	11.4	44.4	2.9	6.7	0.0	21.7	16.7
	S108N	Mutant	100	100	84.7	100	100	100	93.6	100
		Mixed	0.0	0.0	8.2	0.0	0.0	0.0	4.3	0.0
		Wild-type	0.0	0.0	7.1	0.0	0.0	0.0	2.1	0.0
Pfdhps	A437G	Mutant	82.8	84.1	78.5	97.3	92.0	97.1	197.3	76.7
		Mixed	20.7	25.0	25.8	0.0	16.0	0.0	13.5	2.3
		Wild-type	3.4	9.1	2.2	2.7	12.0	2.9	0.0	2.3
	K540E	Mutant	71.0	65.9	67.7	97.3	60.0	97.1	69.6	95.3
		Mixed	19.4	25.0	29.3	0.0	30.0	0.0	17.4	2.3
		Wild-type	9.7	9.1	3.0	2.7	10.0	2.9	13.0	2.3

Supplemental Table 5

PfKelch13 mutations observed in this study

Codon position	Codon (nucleotide) change	*N*	Collection year	Genbank accession number (sample ID)
448*	I (ata) → M (atg)	1	2015	MF345252 (Kombewa-2015-KM027b)
457*	L (tta) → I (ata)	1	2008	MF345158 (Kombewa-2008-KM2027)
469*	C (tgc) → W (tgg)	1	2015	MF345232 (Kakamega-2015-KK005b)
469	C (tgc) → C (tgt)	3	2003	MF344998 (Kombewa-2003-KM019)
			2003	MF345019 (Kombewa-2003-KM170)
			2015	MF345242 (Kakamega-2015-KK079b)
474	T (aca) → T (act)	1	2003	MF344983 (Kakamega-2003-IG1093)
490*	N (aat) → S (agt)	1	2005	MF345070 (Kombewa-2005-KM012b)
507	E (gaa)→ E (gag)	1	2008	MF345154 (Kombewa-2008-KM1002)
513*	R (cgt) → S (agt)	1	2015	MF345225 (Kakamega-2015-IG163b)
522*	S (agt) → C (tgt)	1	2015	MF345266 (Kombewa-2015-KM099b)
548	G (ggc) → G (ggt)	1	2008	MF345148 (Kombewa-2008-KM2069b)
554*	A (gca) →S (tca)	1	2015	MF345286 (Kombewa-2015-KM323b)
569*	A (gca) → S (tca)	1	2003	MF344978 (Kakamega-2003-IG444)
578*	A (gct) → S (tct)	4	2008	MF345156 (Kombewa-2008-KM2005)
			2015	MF345261 (Kombewa-2015-KM064b)
			2015	MF345187 (Kakamega-2015-KK010)
			2015	MF345231 (Kakaemga-2015-KK004)
590*	I (att) → F (ttt)	1	2015	MF345278 (Kombewa-2015-KM214)
597	R (aga) → R (agg)	1	2015	MF345217 (Kakamega-2015-IG042b)
612*	E (gaa) → D (gat)	3	2015	MF345246 (Kombewa-2015-KM106b)
			2015	MF345269 (Kombewa-2015-KM107)
			2015	MF345270 (Kombewa-2015-KM108)

Asterisk denotes nonsynonymous mutation.
